# Atypical oral candidiasis in a psoriatic patient during targeted immunotherapy with an interleukin 17 inhibitor (secukinumab)

**DOI:** 10.1186/s12903-021-01653-6

**Published:** 2021-06-08

**Authors:** Bruna Lavinas Sayed Picciani, Arkadiusz Dziedzic, Juliana Tristão Werneck, Marcello Alves Marinho, Thaylla Núñez Amin Dick, Nara Regina Quintanilha, Eliane Pedra Dias

**Affiliations:** 1 Postgraduate Program in Pathology, School of Medicine, Universidade Federal Fluminense, Rio de Janeiro, Brazil; 2 Postgraduate Program in Dentistry, School of Dentistry, Universidade Federal Fluminense, Nova Friburgo, Rio de Janeiro, Brazil; 3 Dental Center for Patients with Special Needs, Instituto Rir, Rio de Janeiro, Brazil; 4 Department of Conservative Dentistry with Endodontics, Medical University of Silesia, Katowice, Poland; 5 Medical Clinic Service, Hospital Antônio Pedro, School of Medicine, Universidade Federal Fluminense, Rio de Janeiro, Brazil

**Keywords:** Oral candidiasis, Secukinumab, Psoriasis, Interleukin 17 inhibitor (anti IL-17)

## Abstract

**Background:**

Secukinumab is a human monoclonal antibody immunoglobulin that neutralises interleukin (IL)-17A, and as such, is effective in the treatment of psoriasis. However, as IL-17A is essential in protection against fungal infections, patients treated with this drug may develop candidiasis. This report presents a case of atypical oral candidiasis occurring during targeted drug immunotherapy with an interleukin 17 (IL-17) inhibitor (secukinumab), with the aim of emphasisinge the necessity of periodical oral health assessment and monitoring. It provides a rational clinical approach to therapeutic protocol in the treatment of side effects associated with novel medications for autoimmune diseases.

**Case presentation:**

Symptomatic tongue lesions were observed in a 50-year-old female patient on a monthly systemic treatment of 300 mg of secukinumab, which appeared after 60 days of using the medication. Two inconclusive biopsies and an unsuccessful application of oral corticosteroids made the diagnostic process challenging. Papillae on the back of the tongue were atrophied, forming a well-defined erythema and white non-detachable plaques on the lateral border of the tongue. Cytopathological and histopathological exam results were compatible with a diagnosis of oral candidiasis. Topical antifungal medication led to subsequent regression of the tongue lesions. During asymptomatic period and follow up for 7 months, a reduced monthly dose 150 mg of secukinumab was administered.

**Conclusions:**

Patients undergoing treatment with IL-17 blockers, such as secukinumab, should be carefully monitored in order to avoid oral side effects resulting from the use of this medication.

## Background

IL-17-mediated immunity to opportunistic fungal pathogens can be severely impaired as a result of the therapeutic effects of novel targeted drugs. Secukinumab is a human monoclonal antibody that targets and blocks interleukin 17A (IL-17A), exhibiting great efficacy in the treatment of psoriasis and psoriatic arthritis [1, 2]. As IL-17A plays a pivotal role in host protection against fungal infections, patients treated with secukinumab may develop oral candidiasis [3]. Such association has been noted in chronic mucocutaneous diseases in humans, resulting in the persistence or recurrence of Candida infections with mutations of IL-17-related genes [4]. These infections are dependent on 300 mg and 150 mg doses of secukinumab [5].

Oral candidiasis is the most common opportunistic fungal infection in humans, its prevalence has been increasing due to widespread use of antibiotics and the rapid development of novel immunosuppressive drugs. It comprises several clinical forms. Chronic hyperplastic candidiasis, a rare and atypical one, can be difficult to diagnose [6, 7]. Thus, a clinical examination may be insufficient, and the use of reliable methods, such as cytopathological and histopathological assessments, is necessary [8–10]. Despite sever reports of oral candidiasis in patients taking secukinumab, their occurrence in the literature is still sparse [10–12]. Moreover, we did not find any cases of chronic hyperplastic candidiasis confirmed by cytopathological examination in these patients. Therefore, the aim of this study is to report a case of oral candidiasis during treatment with secukinumab and to underline the advantages of oral cytopathology in investigating atypical oral candidiasis, as well as to highlight the importance of dentist and dermatologists in monitoring such patients.

## Case presentation

A 50-year-old white female patient was referred to the Oral Medicine Department of Fluminense Federal University due to symptomatic oral mucosa lesions that were causing burning sensations and spontaneous pain. The patient had been diagnosed with an autoimmune condition—psoriasis in childhood and with psoriatic arthritis within for the past 10 years. She had undergone a specific, immuno-modulating pharmacotherapy with IL-17 inhibitor, secukinumab, which had lasted for six months. Interestingly, despite previous treatment options, she showed no response to tumour necrosis factor (TNF) inhibitors, such as adalimumab. What is more, the patient could not tolerate cyclosporine therapy.

Initially, the patient reported a noticeable presence of suspicious and painful lesions on borders and lateral edges of her tongue, which appeared after 60 days of taking secukinumab. Two subsequent, standard inconclusive biopsies were previously carried out on both edges. Topical oral corticosteroids and betamethasone were prescribed, without any substantial improvement or positive treatment outcome. In addition, no cutaneous lesions were observed, and joint symptoms were well controlled by means of the prescribed therapy.

**Fig. 1:**
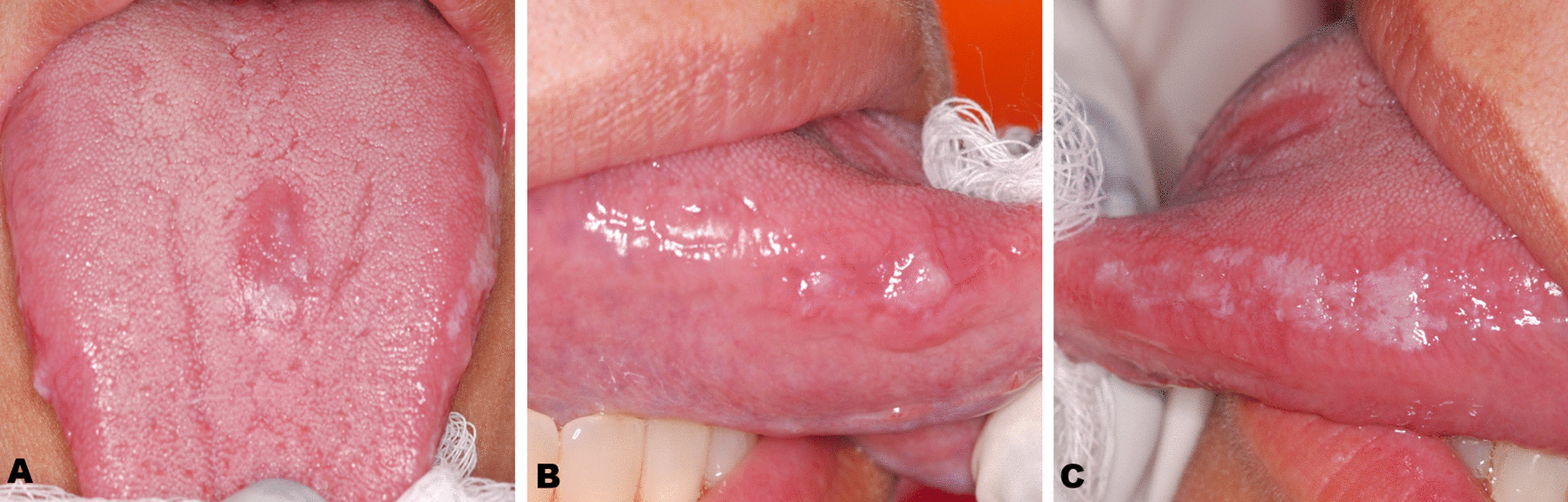
Clinical aspects of oral lesion: **A** Well-defined Erythematous lesion on the back of the tongue. **B**, **C** Non-detachable white plate on the lateral of the tongue

On intraoral examination, atrophy of the papillae on the posterior part of tongue were noted adjacent to a well-defined erythema and white non-detachable plaque on the lateral edges of the tongue (Fig. 1). Non-invasive mini scrapes from both lateral borders and the dorsal surface of the tongue were obtained using a sterile cytobrush (Kolplast®, Brazil). The Papanicolaou staining, periodic acid-Schiff (PAS) technique and cytopathological analysis were performed by an oral pathology specialist at the Pathology Centre of Antônio Pedro University Hospital (Fluminense Federal University, Niterói-RJ, Brazil).

**Fig. 2:**
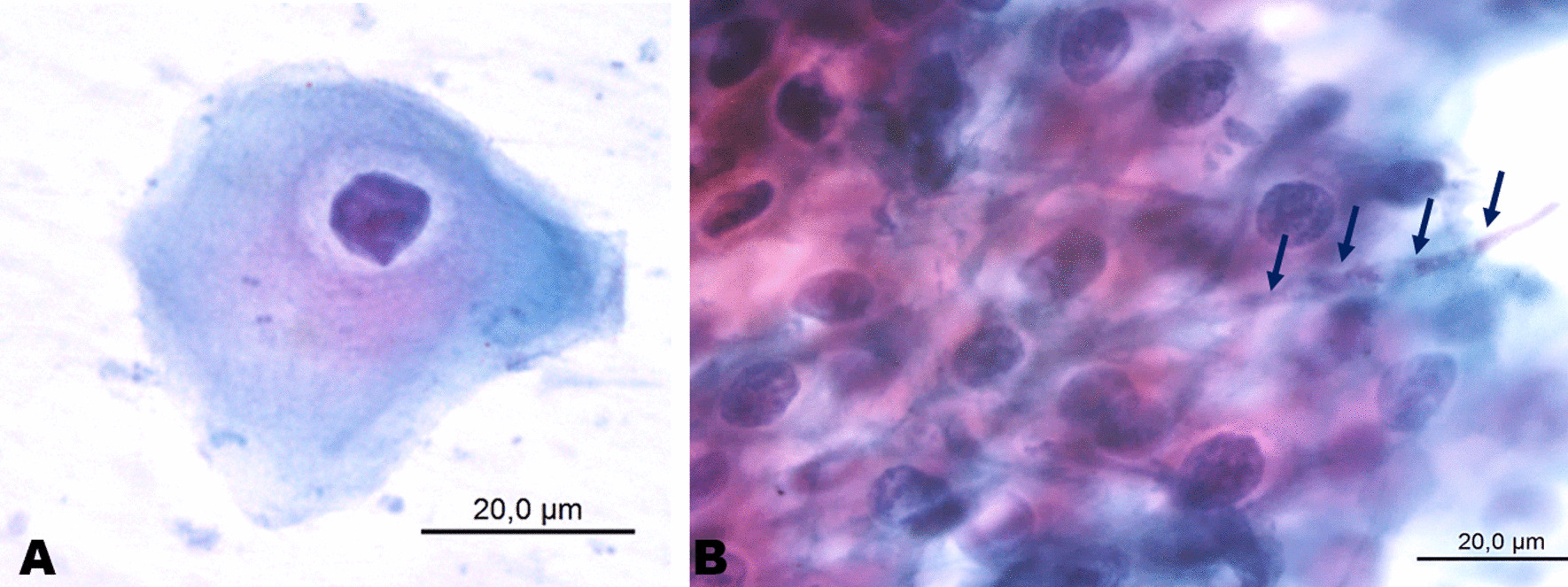
Cytopathological aspects of oral candidiasis: **A** Presence of keratinocytes with volume alterations and nuclear chromatism. **B** *Candida spp*. hyphae permeating the keratinocytes (Papanicolau)

**Fig. 3:**
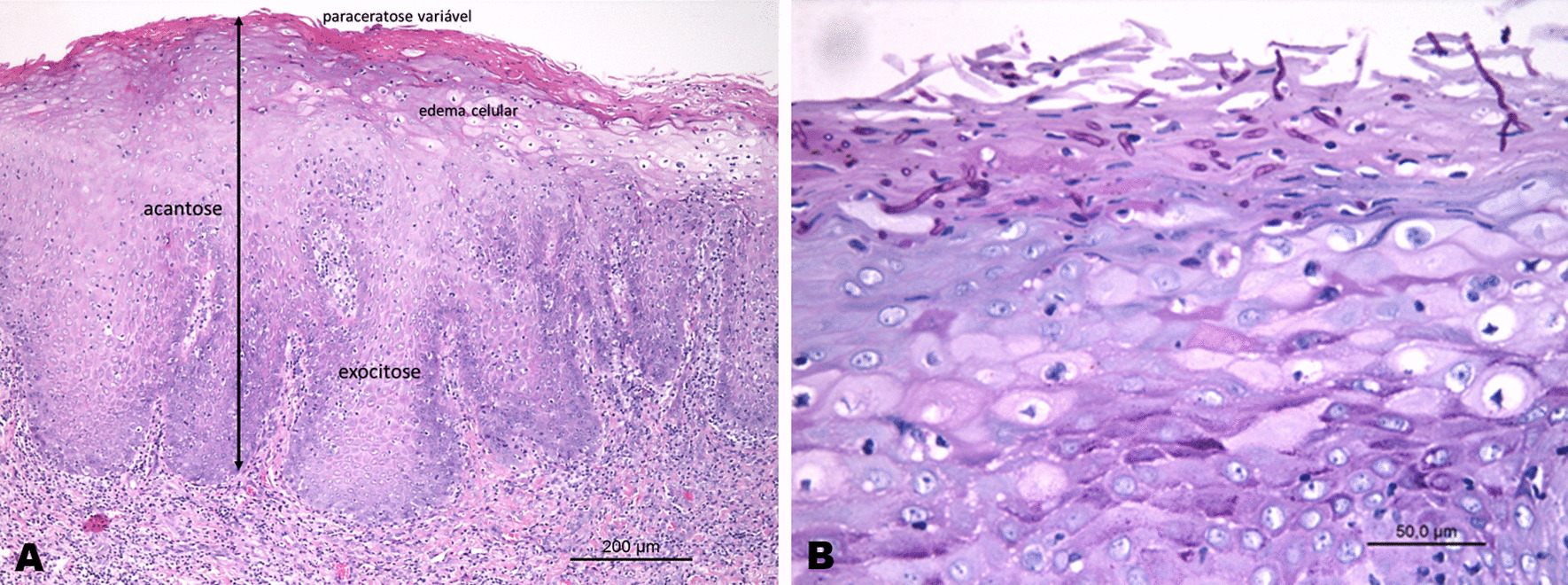
Histopathological aspects of oral candidiasis: **A** Fragment showed Parakeratosis, acanthosis, exocytosis of polymorphonuclear leukocytes, permeating corneal layer of the epithelium and moderately diffused perivascular inflammatory infiltrate (hematoxilina and eosina stain). **B** Periodic acid-Schiff stain showed the presence of *Candida* sp. hyphae permeating the stratum corneum

The cytopathological investigation revealed keratinised clustered cells, often overlapped, with marked parakeratosis, as well as pseudo-hyphae and hyphae of *Candida spp. *(Fig. 2). Interestingly, the subsequent histopathological examination of the biopsy block scraps showed non-characteristic features of parakeratosis, acanthosis, exocytosis of polymorpho-nuclear leukocytes, hyphae of *Candida spp*. permeating the corneal layer of the epithelium and moderately diffuse perivascular inflammatory infiltration (Fig. 3). Based on clinical and laboratory assessment, adefinitive diagnosis of medication-related oral candidiasis was made.

**Fig. 4:**
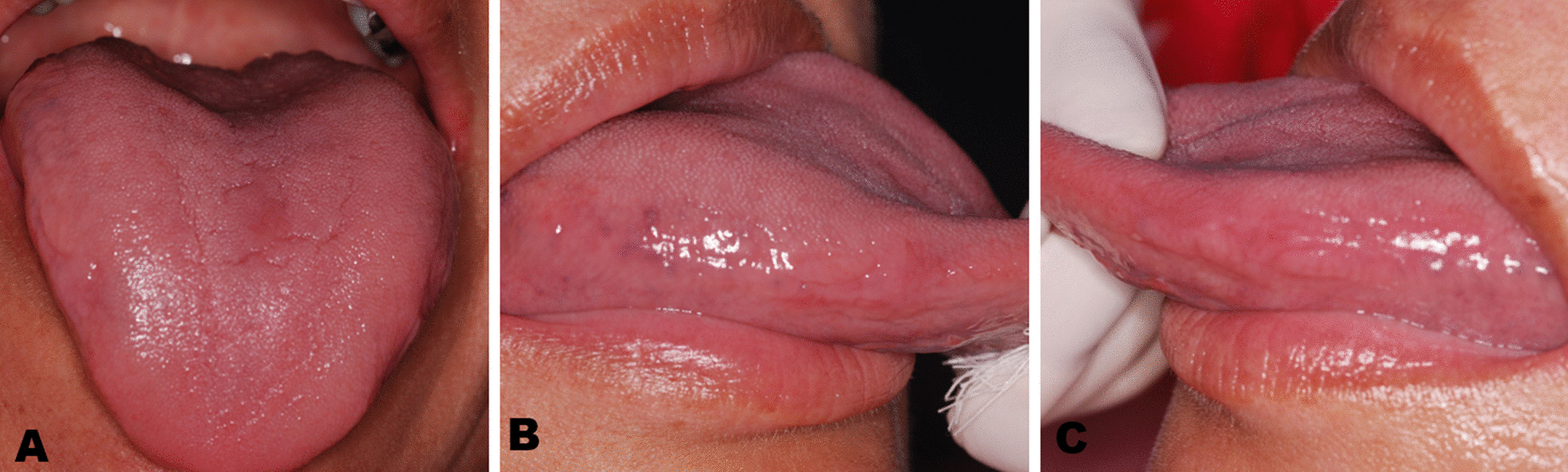
Clinical aspects of oral candidiasis after treatment, showing total regression

A standard therapy of topical miconazole gel, 20 mg four times a day for 30 days was prescribed. The patient was advised to discontinue secukinumab therapy for 30 days, following consultation with a dermatologist. After this period, the symptomatic oral mucosa lesions regressed and subsided completely, which allowed the patient to restart and continue secukinumab therapy at a reduced dose of 150 mg monthly. As at the time of this report, the patient has followed up on the secukinumab for seven months and there has been no recurrence of oral lesion has been observed (Fig. 4).

## Discussion and conclusions

This study documented candida infection occurrence in a psoriatic patient taking 300 mg of secukinumab. From a pathological and biochemical data it appears clear that, an inflammatory mediator: interleukin-17A is directly involved in the development of psoriasis and consequently in psoriatic arthritis. Furthermore, it plays an important role in immunological systemic protection against opportunistic infections, especially Candida *spp.* This bi-directional association is seen in chronic mucocutaneous diseases in humans, resulting in the persistence or recurrence of Candida infections in cases of IL-17-related mutations of genes [4, 13]. In recent systematic review of the risk of infection in patients treated with anti-IL-17A antibodies, including secukinumab, Candida *spp.* infections were reported in 1.7% of patients undergoing targeted immunotherapy [14]. All Candida *spp.* infections that occurred in secukinumab study groups were localized: typically oral or genital candidiasis of mild to moderate severity [15]. By comparison, our patient suffered only from oral candidiasis.

**Fig. 5:**
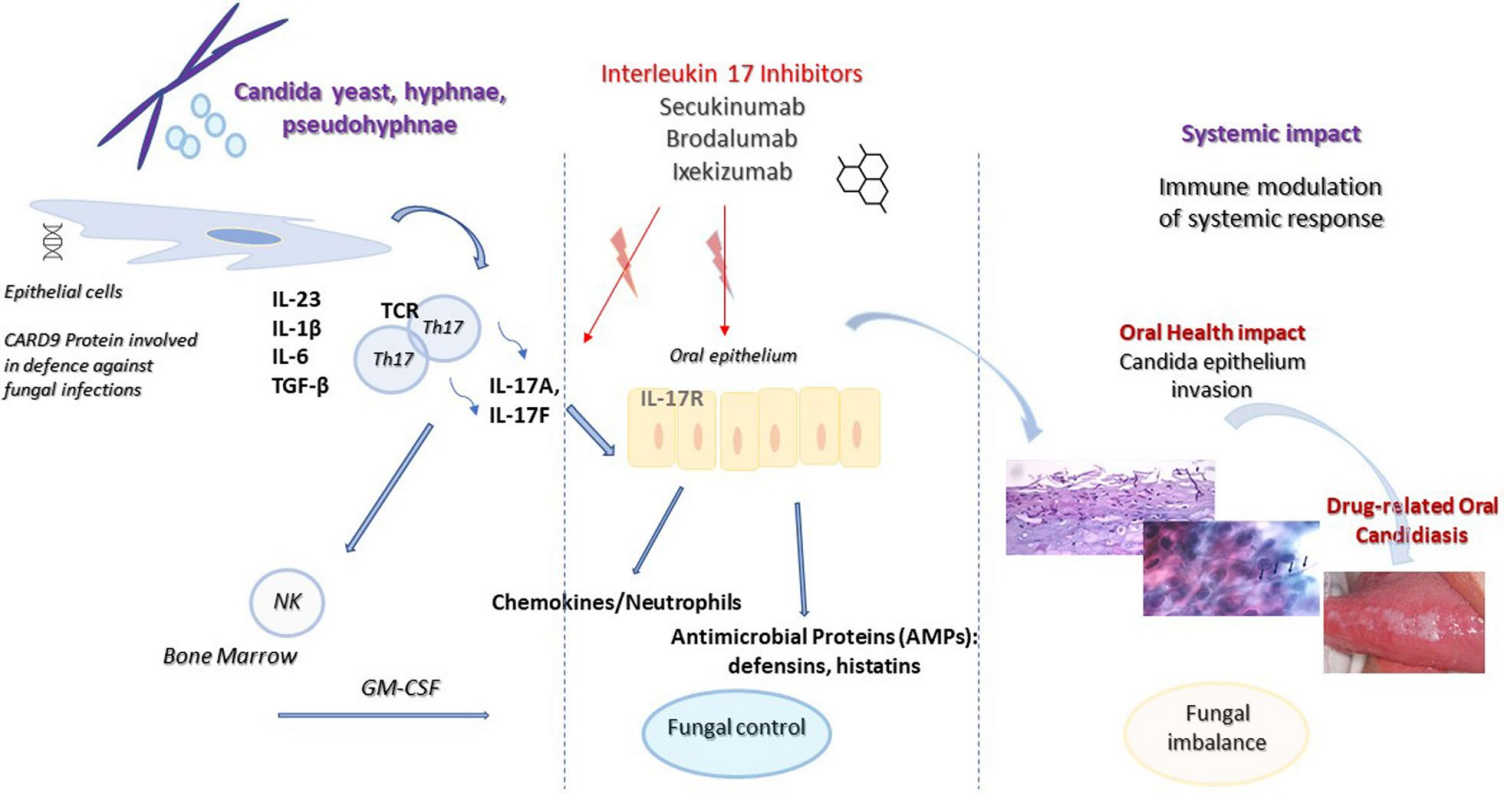
Schematic demonstrating the overall hypothesis that the blocks interleukin (IL)-17A play key roles in oral candidiasis pathogenesis

A variety of predisposing local and systemic factors may lead to Candidas’ transition from a commensal to pathological role. Over the last decade, targeted-drug immunotherapy with selected monoclonal antibodies has been described as a factor predisposing for oral candidiasis (Fig. 5) [9].

Although oral candidiasis is a common opportunistic infection in general, its chronic hyperplastic form is rather uncommon, with frequent occurrences mainly within the buccal mucosa and labial commissure [6, 7]. In our case, the lesions appeared on the lateral border and on the back of the dorsal surface of the tongue, hampering tentative, and even differential diagnosis. Standard cytopathological exam is recommended as a basic and simple test that can enable fast and accurate diagnosis, this was essential for the definition of the reported case [8, 9]. Oral liquid-based cytology too may have considerable potential for detection of lesions [16]. This allows the ruling out other differential diagnoses made, including leukoplakia, oral lichen planus, or non-specific lichenoid reaction, as well as the less common hairy leukoplakia. Furthermore, the diagnosis of oral candidiasis should be supported by histopathologic assessment and characteristic findings. As expected, the IL-17-related oral lesions responded well to therapy and regressed after treatment with conventional topical antifungal therapy. In addition, adjusting the dose of secukinumab to 150 mg seemed to vastly reduce the recurrence of candidiasis. Studiesrevealed that the frequency of Candida infection was higher when 150 mg to 300 mg of secukinumab were administered (0–50%), than when lower doses of 75 mg were used (10%) [14, 15]. According to the results reported in the study of Blauvelt et al., secukinumab administration once weekly until week 4 and again at week 8, plus at week 12 in case of two patients (34%) in the 300 mg secukinumab group resulted in diagnosing them, along with one patient (17%) in the 150 mg secukinumab group, with Candida *spp.* oral infection [15]. Table 1 compares the incidence of oral candidiasis during therapy with IL-17 inhibitors in psoriatic patients with cases of psoriatic arthritis [5, 15, 17–23].

**Table 1:** Incidence of oral candidiasis during interleukin (IL)-17 inhibitors treatment of psoriatic patients and psoriatic arthritis

References	Disease	Number of patient	Treatment regimen	% (n) Oral candidiasis
Langley et al. [5]	Psoriasis	349	Secukinumab 300 mg	2% (7)
		353	Secukinumab 150 mg weeks 12–52	0.8% (3)
Blauvelt et al. [15]	Psoriasis	59	Secukinumab 300 mg 12 weeks	1.7% (1)
Thaçi et al. [17]	Psoriasis	21	Secukinumab 300 mg 36 weeks	4.8% (1)
Mease et al. [18]	Psoriatic Arthritis	295	Secukinumab 150 mg	1.4% (4)
		292	Secukinumab 75 mg 52 weeks	1.4% (4)
McInnes et al. [19]	Psoriatic Arthritis	100	Secukinumab 300 mg	2% (2)
		100	Secukinumab 150 mg	2% (2)
		99	Secukinumab 75 mg 52 weeks	1% (1)
Papp et al. [20]	Psoriasis	181	Brodalumab 210 mg Every 2 week	2.8% (5)
Nakagawa et al. [21]	Psoriasis	37	Brodalumab 210 mg 12 weeks	2.7% (1)
Yamasaki et al. [22]	Psoriasis	30	Brodalumab 140 mg 12 weeks	3.3% (1)
Gordon et al. [23]	Psoriasis	3736	Ixekizumab 80 mg 60 weeks	1.7% (63)

Considering a wider population, a well-structured randomised control study on the oral health-related side effects of long-term treatment with IL-17 inhibitors seems to be essential to understanding the risk rate for Candida *spp.* opportunistic infections in patients with autoimmune diseases. Eventually, antifungal and cytoprotective prophylactic measures may be implemented as a standard protocol, protecting patients with a previous history of recurrent oral candidiasis, medical comorbidities, or other predisposing factors.

It is predicted that a growing number of novel therapies modulating immune system will inevitably lead to the more frequent prevalence of atypical oral lesions associated with specific mechanisms of interaction with biological structures. Oral medicine specialists should expect an increasing number of atypical oral manifestations in the systemic treatment of common autoimmune diseases, such as: arthritis, psoriasis, etc. Specialists responsible for prescribing these targeted drugs are obliged to provide sufficient information regarding less common side effects of interleukin inhibitors, stressing the need for regular dental assessments aimed at early diagnosis of adverse effects of pharmacological therapy.

In conclusion, novel targeted drug-related oral candidiasis can occur in patients with autoimmune conditions who are on prolonged therapy with IL-17 blockers. Persons receiving specific prolonged monoclonal antibody treatment should be carefully monitored by a dental practitioner and dermatologist to prevent oral health complications resulting from the use of immunity-modulating drugs. The reduction in the dosage of the interleukin inhibitor provides a rational approach, preventing oral manifestation of drug-related side effects.

## Data Availability

Not applicable. This is a case report without more data and materials.

## Abbreviations

IL-17: Interleukin 17
TNF: Tumour necrosis factor
PAS: Periodic acid-Schiff
